# Prevalence and network structure of post-traumatic stress symptoms and their association with suicidality among Chinese mental health professionals immediately following the end of China’s Dynamic Zero-COVID Policy: a national survey

**DOI:** 10.1038/s41398-023-02680-3

**Published:** 2023-12-15

**Authors:** Pan Chen, Ling Zhang, Yuan Feng, Feng-Rong An, Zhaohui Su, Teris Cheung, Ka-In Lok, Gabor S. Ungvari, Todd Jackson, Yu-Tao Xiang, Qinge Zhang

**Affiliations:** 1 Unit of Psychiatry, Department of Public Health and Medicinal Administration, & Institute of Translational Medicine, Faculty of Health Sciences, University of Macau, Macao SAR, China; 2 Centre for Cognitive and Brain Sciences, University of Macau, Macao SAR, China; 3 The National Clinical Research Center for Mental Disorders & Beijing Key Laboratory of Mental Disorders, Beijing Anding Hospital & the Advanced Innovation Center for Human Brain Protection, Capital Medical University, Beijing, China; 4 School of Public Health, Southeast University, Nanjing, China; 5 School of Nursing, Hong Kong Polytechnic University, Hong Kong SAR, China; 6 Faculty of Health Sciences and Sports, Macao Polytechnic University, Macao SAR, China; 7 University of Notre Dame Australia, Fremantle, WA Australia; 8 Division of Psychiatry, School of Medicine, University of Western Australia/Graylands Hospital, Perth, WA Australia; 9 Department of Psychology, University of Macau, Macao SAR, China

**Keywords:** Psychiatric disorders, Scientific community

## Abstract

Studies on post-traumatic stress symptoms (PTSS) among mental health professionals (MHPs) are limited, particularly since restrictions due to coronavirus disease (COVID-19) have been lifted such as the recent termination of China’s Dynamic Zero-COVID Policy. The current study filled this gap by exploring the prevalence, correlates, and network structure of PTSS as well as its association with suicidality from a network analysis perspective. A cross-sectional, national survey was conducted using a convenience sampling method on MHPs between January 22 and February 10, 2023. PTSS were assessed using the Post-Traumatic Stress Disorder Checklist-Civilian version, while suicidality was assessed using standardized questions related to ideation, plans, and attempts. Univariate and multivariate analyses examined correlates of PTSS. Network analysis explored the structure of PTSS and suicidality. The centrality index of “Expected influence” was used to identify the most central symptoms in the network, reflecting the relative importance of each node in the network. The “flow” function was adopted to identify specific symptoms that were directly associated with suicidality. A total of 10,647 MHPs were included. The overall rates of PTSS and suicidality were 6.7% (*n* = 715; 95% CI = 6.2–7.2%) and 7.7% (*n* = 821; 95% CI = 7.2–8.2%), respectively. Being married (OR = 1.523; *P* < 0.001), quarantine experience (OR = 1.288; *P* < 0.001), suicidality (OR = 3.750; *P* < 0.001) and more severe depressive symptoms (OR = 1.229; *P* < 0.001) were correlates of more PTSS. Additionally, higher economic status (e.g., good vs. poor: OR = 0.324; *P* = 0.001) and health status (e.g., good vs. poor: OR = 0.456; *P* < 0.001) were correlates of reduced PTSS. PCL6 (“Avoiding thoughts”; EI = 1.189), PCL7 (“Avoiding reminders”; EI = 1.157), and PCL11 (“Feeling emotionally numb”; EI = 1.074) had the highest centrality, while PCL12 (“Negative belief”), PCL 16 (“Hypervigilance”) and PCL 14 (“Irritability”) had the strongest direct, positive associations with suicidality. A high prevalence of lingering PTSS was found among MHPs immediately after China’s “Dynamic Zero-COVID Policy” was terminated. Avoidance and hyper-arousal symptoms should be monitored among at-risk MHPs after the COVID-19 pandemic and serve as potential targets for the prevention and treatment of PTSS in this population.

## Introduction

The outbreak of the novel coronavirus disease (COVID-19) in 2019 brought unprecedented challenges to countries across the globe. In response, the government of China implemented the “Dynamic Zero-COVID Policy”, as an effective strategy to control the transmission of COVID-19 for a short period at a lower cost [1]. Despite efforts to minimize negative effects on the economy, society, and daily lives of people [2, 3], strict measures from the policy (i.e. unpredictable and sudden lockdowns, constant nucleic testing and restrictions on travel or traffic) had unintended consequences [1], including increased risk for depression, anxiety, insomnia and post-traumatic stress disorder (PTSD) [4, 5]. However, as morbidity and mortality risks of COVID-19 remitted, during December 2022 China relaxed its restrictions and terminated the “Dynamic Zero-COVID Policy” [6]. After the COVID-19 pandemic had reached its peak over the past three years [7], China’s reopening policy was launched and has attracted considerable attention in relation to lingering post-pandemic effects.

PTSD is a serious psychiatric condition caused by unpredictable traumatic events involving actual or threatened death, accidents, physical assault, sexual violence, natural disasters or emergent public health events [8] and is characterized by a set of symptoms that reflect intrusion/re-experiencing, avoidance, negative cognitions, negative mood, and hyper-arousal [9]. PTSD is associated with increased anxiety levels, memory impairment, interpersonal communication problems, decreased work quality, physical health comorbidities, and exacerbated risk for suicidal behavior [10]. The occurrence of post-traumatic stress symptoms (PTSS) can be triggered by direct exposure to traumatic events as well as witnessing, indirect exposure through effects on close family members or friends, and repeated or extreme exposure [8]. The COVID-19 pandemic and responses to prevent or control its impact can also increase traumatic stress reactions.

Apart from infected patients and their family members, the psychological burden of COVID-19 has been especially high among healthcare workers (HCWs) [11]. As reported for previous infectious diseases, relatively high levels of PTSS were experienced by hospital HCWs at risk of contracting severe acute respiratory syndrome (SARS) within 1–3 years after the SARS outbreak [12]. The epidemiology of PTSS among HCWs has also been estimated during the COVID-19 pandemic, with rates varying from 13% to 25.6% [11, 13–15]. Similarly, mental health professionals (MHPs) have been vulnerable to suffering from PTSS during the COVID-19 pandemic. In China, MHP risk may be partially due to historically marginalized psychiatric services [6, 16], insufficient training in response to emergent public health events and the increased risk of exposure to infected psychiatric inpatients [17]. Various risk factors including female gender [18], young age [15], less work experience [19], personal quarantine experience during the pandemic [14], economic burdens [19], and heavy workloads [18] may also correlate with increased PTSS within this sector of China’s workforce.

Traditional conceptualizations of psychopathology have treated disorders or syndromes as sets of symptoms with equal weights. However, such approaches tend to ignore dynamics and interconnections between symptoms [20]. Network analysis has emerged as a novel method for understanding interrelationships between symptoms and provides an alternative that addresses limitations of traditional latent factor analysis approaches [21]. In a symptom network model, the most central symptoms, or symptoms that have the strongest associations with other symptoms, are considered to be viable targets for interventions used to treat psychiatric syndromes [22, 23].

To date, select network research of PTSS has examined connections between specific PTSS and quality of life (QOL) among MHPs during the initial stage of the COVID-19 pandemic [24]. Whereas avoidance and numbing symptoms (i.e. “Avoidance of thoughts”, “Avoidance of reminders” and “Emotionally numb”) were identified as the most central symptoms in the PTSS network, hyperarousal symptoms (i.e. “Sleep disturbances”, “Irritability” and “Difficulty concentrating”) had the strongest associations with QOL. Despite such data, it is not clear whether or how rates, correlates, or network structures of PTSS have changed among MHPs since China’s Dynamic Zero-COVID Policy ended. Understanding the prevalence and predictors of PTSS as well as the most influential PTSS associated with suicidality among MHPs is critical for developing effective measures to prevent and alleviate lingering negative effects of PTSS in this population. Therefore, this study was designed to investigate the prevalence and correlates of PTSS among MHPs, generate a post- Dynamic Zero-COVID Policy network model of PTSS, and identify particular PTSS related to increased suicidality within this population.

## Methods

### Study design and participants

A cross-sectional, national survey was conducted by panel members of the Psychiatry Branch, Chinese Nursing Association and the Chinese Society of Psychiatry between January 22 and February 10, 2023 (i.e., immediately after the China’s Dynamic Zero-COVID Policy ceased) using a snowball convenience sampling method. Following previous studies [25–27], to avoid the potential risk of infection during the COVID-19 pandemic, we conducted an online survey with WeChat-based Questionnaire Star instead of traditional face-to-face interviews. WeChat is a popular communication program that is widely used in clinical practice and continuing educational activities. In addition, all health professionals in China were mandated to report health status each day during the COVID-19 pandemic and were, presumably, WeChat users [28, 29]. The QuestionnaireStar program is a widely used epidemiological survey tool in China [30]. A Quick Response Code (QR code) linked to the study invitation and a questionnaire generated by the WeChat-based Questionnaire Star program were distributed to all public psychiatric hospitals nationwide. Eligible volunteers were (1) adults aged 18 years or above, (2) MHPs (e.g. psychiatrists, nurses or technicians) working in psychiatric hospitals or psychiatric departments of general hospitals in China during the COVID-19 pandemic and (3) able to understand Chinese and provide written informed consent. There were no specific exclusion criteria in this study. The study protocol was approved by the Ethics Committee of the Beijing Anding Hospital, China.

### Measures

Socio-demographic data collected included age, gender, marital status, educational level, clinical work duration (years), living status, perceived economic and health status, and COVID-19 infection and quarantine experience during the pandemic.

PTSS were assessed using a Chinese validation of the Post-Traumatic Stress Disorder Checklist-Civilian version (PCL-C) [31, 32]. The PCL-C is a standardized self-report rating scale for PTSD that corresponds to the key symptoms of PTSD based on DSM-IV criteria [33]. The stressful event was set as COVID-19 and COVID-19-related prevention and control measures adopted in China (i.e., Dynamic Zero-COVID Policy). The PCL-C consists of 17 items covering three dimensions: Intrusion (5 items), Avoidance/Numbing (7 items), and Hyperarousal (5 items) [34]. Each item was rated on a five-point Likert scale from 1 (‘not at all’) to 5 (‘extremely’). Total scores ranged from 17 to 85, with higher values indicating more severe PTSS. A score between 38 and 49 was defined as “having some degree of PTSS”, while a total score of ≥50 was defined as “having significant PTSS” [32]. In this study, the cut-off value of 38 was used to identify the participants with PTSS.

Depressive symptoms were measured with the validated Chinese version of the nine-item Patient Health Questionnaire (PHQ-9) [35, 36]. Each item was rated on a 4-point frequency scale from 0 (‘not at all’) to 3 (‘nearly every day’). Total scores ranged from 0 to 27, with higher values indicating more severe depressive symptoms. In addition, suicidality during the past week was assessed by three standardized questions that queried whether participants ever had suicide ideation (“Have you ever seriously thought about committing suicide?”), suicide plans (“Have you ever made a plan for committing suicide?”) or suicide attempts (“Have you ever attempted suicide?”) [37]. Participants reporting any of these three experiences were classified as “having suicidality”.

### Statistical analysis

#### Univariate and multivariate analyses

Univariate and multivariate analyses were performed using SPSS version 26.0 (SPSS Inc., Chicago, Illinois, USA). One-sample Kolmogorov-Smirnov tests were used to test normality distributions of continuous variables. Comparisons of sociodemographic and clinical variables between participants with PTSS versus those without PTSS were conducted using independent sample *t*-tests or Mann-Whitney *U* tests for continuous variables and Chi-square tests for categorical variables, as appropriate. A binary logistic regression analysis was performed to examine independent correlates of PTSS; having versus not having PTSS was the dependent variable and univariate measures on which PTSS and non-PTSS subgroups had significant differences in were included as independent variables based on the “Enter” method. Significant statistical differences were set at *P* < 0.05 (two-tailed).

#### Network structure of PTSS

Network structure analysis was conducted using R software (version 4.2.2) [38]. For the PTSS network, a Graphical Gaussian Model (GGM) with graphic least absolute shrinkage and selection operator (LASSO) and an Extended Bayesian Information Criterion (EBIC) model were applied [39]. Network estimation was assessed using the “estimateNetwork” function in R “bootnet” package with “EBICglasso” method and visualized by “qgraph” [40] and “ggplot2” packages [41]. Nodes represented individual PTSS and edges represented correlations between symptoms. Thicker edges represented stronger correlations, green edges reflected positive correlations and red edges reflected negative correlations.

The network properties of each node were evaluated using indices of expected influence (EI) and predictability that were calculated by “qgraph” [40] and “mgm” [42], respectively. EI referred to the sum of a node’s connections, reflecting the relative importance of a node in the network [43]. Predictability was an absolute measure of the interconnectedness of a given node in the network, reflecting shared variance of a given node with its neighboring nodes [42, 44].

The stability and accuracy of the network model were evaluated using the “bootnet” package [39]. A correlation stability coefficient (CS-C), calculated by a case-drop bootstrapping method, was used to evaluate network stability; a minimum value of 0.25 was considered to reflect a stable network though a value of 0.5 was preferable. Accuracy was estimated based on bootstrapped 95% confidence intervals (CIs) of edge weights, with narrower ranges indicating a more trustworthy network [39]. We also performed a bootstrapped difference test between the weights of edge pairs [39]. Finally, because previous studies found significant associations between quarantine experiences and PTSS [45, 46], the overall connectivity and network structure of PTSS networks based on quarantined versus non-quarantined samples were compared using Network Comparison Tests (NCT) via the “Network Comparison Test” package [47].

#### The association between PTSS and suicidality

Regarding the relationship between suicidality and individual PTSS in the network model, a Mixed Graphical Model (MGM) was estimated by using the R “bootnet” package with “mgm” as the estimation method. In addition, the “flow” function in R package “qgraph” was applied to clarify the network structure [40].

## Results

### Participant characteristics

Of 11,760 invited cohorts, 10,647 MHPs met the study entry criteria and completed the assessment for a participation rate of 98.0%. Demographic and clinical characteristics of participants are shown in Table 1. MHPs included psychiatrists (581; 5.5%), nurses (9717; 91.3%), and other relevant professionals such as technicians and clinical psychologists (349; 3.3%). The mean age of participants was 34.85 years (SD = 8.395 years) and 18.0% (*n* = 1920) were men. Most participants had a “college or above” education level (*n* = 10,809; 94.8%) and were married (*n* = 7722; 72.5%).

**Table 1:** Demographic and clinical characteristics of the study sample (*N* = 10,647).

Variables	Total (*N* = 10,647)		Without PTSS (*N* = 9932)		With PTSS (*N* = 715)		Univariable analysis		
	*n*	%	*n*	%	*N*	%	*x*^2^	*df*	*p*
Male	1920	18.0	1755	17.7	165	23.1	13.191	1	**<0.001**
College and above	10,089	94.8	9429	94.9	660	92.3	9.275	1	**0.002**
Married	7722	72.5	7174	72.2	548	76.6	6.517	1	**0.011**
Living with others	9454	88.8	8815	88.8	639	89.4	0.255	1	0.613
Occupation type									
Psychiatrists	581	5.5	540	5.4	41	5.7	0.652	2	0.722
Nurses	9717	91.3	9063	91.3	654	91.5			
Other professionals	349	3.3	329	3.3	20	2.8			
Perceived economic status									
Poor	1163	10.9	964	9.7	199	27.8	238.773	2	**<0.001**
Fair	8826	82.9	8323	83.8	503	70.3			
Good	658	6.2	645	6.5	13	1.8			
Perceived health status									
Poor	698	6.6	498	5.0	200	28.0	633.999	2	**<0.001**
Fair	7559	71.0	7085	71.3	474	66.3			
Good	2390	22.4	2349	23.7	41	5.7			
Having COVID-19 infection since 2019	9858	92.6	9183	92.5	675	94.4	3.685	1	0.055
At least 1-week quarantine experience during the COVID-19 pandemic	5873	55.2	5403	54.4	470	65.7	34.643	1	**<0.001**
Any suicidality during the past week	821	7.7	530	5.3	291	40.7	1172.057	1	**<0.001**
	Mean	*SD*	Mean	*SD*	Mean	*SD*	*Z*	*df*	*p*
Age (years)	34.85	8.395	34.76	8.403	36.08	8.185	−4.905	-	**<0.001**
Working length (years)	12.68	9.165	12.59	9.185	13.83	8.809	−4.838	-	**<0.001**
PHQ-9 total	5.27	5.384	4.64	4.710	14.16	6.232	−35.411	-	**<0.001**

Bolded values: <0.05.

*df* degree of freedom, *PTSS* post-traumatic stress symptoms, *PHQ-9* Patient Health Questionnaire-9 items, *SD* standard deviation, *COVID-19* corona virus disease.

### Prevalence and correlates of PTSS

The overall prevalence of PTSS (i.e., PCL-17 total score ≥ 38) was 6.7% (*n* = 715; 95% CI = 6.2–7.2%) and included 304 (2.9%) participants who had significant PTSS (PCL-17 total score ≥ 50). The overall prevalence of suicidality was 7.7% (*n* = 821; 95% CI = 7.2–8.2%) in the sample. As shown in Table 1, compared with the non-PTSS subgroup, the PTSS subgroup was more likely to be older (*P* < 0.001), male (*P* < 0.001), married (*P* = 0.011), to have worked longer in clinical settings (*P* < 0.001), have a poorer perceived economic status (*P* < 0.001) and poorer perceived health status (*P* < 0.001), have had at least a 1-week quarantine experience during the COVID-19 pandemic (*P* < 0.001), have experienced suicidality during the past week (*P* < 0.001) and report a significantly higher mean PHQ-9 score (*P* < 0.001). Moreover, participants with PTSS were less likely to have an education level of college and above (*P* = 0.002). There was no significant difference in PTSS prevalence between psychiatrists, nurses and other professionals (*P* = 0.722).

Table 2 presents the binary logistic regression analysis results. Participants who were married (OR = 1.523; *P* < 0.001), had at least 1-week quarantine experience during the COVID-19 pandemic (OR = 1.288; *P* < 0.001), experienced suicidality during the past week (OR = 3.750; *P* < 0.001) and reported more severe depressive symptoms (OR = 1.229; *P* < 0.001) had a significantly higher risk for membership in the PTSS group. Additionally, participants who had a better economic status (e.g., good vs. poor: OR = 0.324; *P* = 0.001; fair vs. poor: OR = 0.710; *P* = 0.006) and better health status (e.g., good vs. poor: OR = 0.456; *P* < 0.001; fair vs. poor: OR = 0.456; *P* < 0.001) had a significantly lower risk of membership in the PTSS group.

**Table 2:** Independent correlates of PTSS among Chinese mental health professionals (*N* = 10,647).

Variables	Multiple logistic regression analysis		
	*p*	*OR*	95% *CI*
Age (years)	0.088	1.025	0.996–1.054
Male gender	0.179	0.171	0.930–1.475
College and above	0.063	0.709	0.493–1.019
Married marital status	**0.001**	**1.523**	1.201–1.933
Working length (years)	0.332	0.988	0.963–1.013
Perceived economic status	**-**	-	-
Poor	**-**	-	-
Fair	**0.006**	**0.710**	0.557–0.905
Good	**0.001**	**0.324**	0.165–0.638
Perceived health status	**-**	**-**	-
Poor	**-**	**-**	-
Fair	**<0.001**	**0.612**	0.477–0.784
Good	**<0.001**	**0.456**	0.300–0.692
At least 1-week quarantine experience during the COVID-19 pandemic	**0.009**	**1.288**	1.065–1.557
Any suicidality during the past week	**<0.001**	**3.750**	3.039–4.627
PHQ-9 total	**<0.001**	**1.229**	1.209–1.248

Bolded values: <0.05.

*CI* confidence interval, *OR* odds ratio, *COVID-19* corona virus disease.

### Network structure of PTSS

**Fig. 1:**
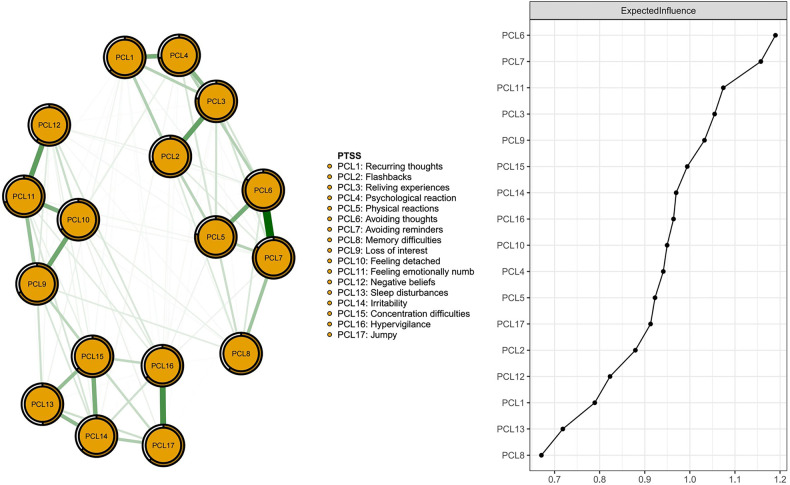
PTSS network among Chinese MHPs. Left: Network structure of PTSS; Right: Centrality index of EI for each node.

As shown in Fig. 1, the three nodes with the highest centrality in the network structure of PTSS were PCL6 (“Avoiding thoughts”), PCL7 (“Avoiding reminders”), and PCL11 (“Feeling emotionally numb”). These nodes were all members of the “Avoidance/Numbing” dimension. The mean predictability of the 17 nodes was 0.682, suggesting an average of 68.2% of the variance in each node could be accounted for by its neighboring nodes in the model. Descriptive information of each PTSS is shown in Table S1.

Figure S1 illustrates the network stability results. The CS-C was 0.75, indicating a stable network. Bootstrapped 95% CIs for estimated edge weights with a narrow range suggested that the network was reliable and stable (Fig. S2). Most comparisons of edge weights were statistically significant based on bootstrapped difference tests (Fig. S3).

**Fig. 2:**
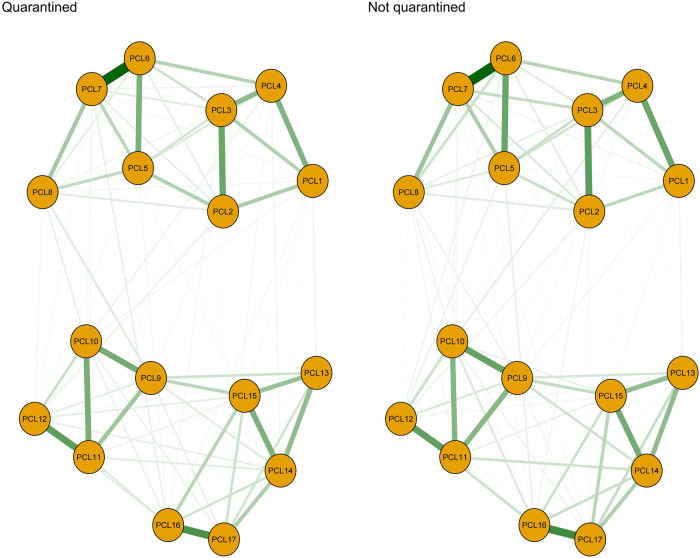
Comparisons of network structures of PTSS. Left: Network structure of PTSS in samples with at least 1-week quarantine experience (*n* = 5873); Right: Network structure of PTSS in samples without quarantine experience (*n* = 4774).

Figure 2 and S4 show PTSS network structures and network properties between quarantined versus non-quarantined subgroups. NCT results indicated there were no significant differences in overall connectivity (*S* = 0.048; *P* = 0.543) or network structure (*M* = 0.07; *P* = 0.943) between the two PTSS networks.

### The association between PTSS and suicidality

**Fig. 3:**
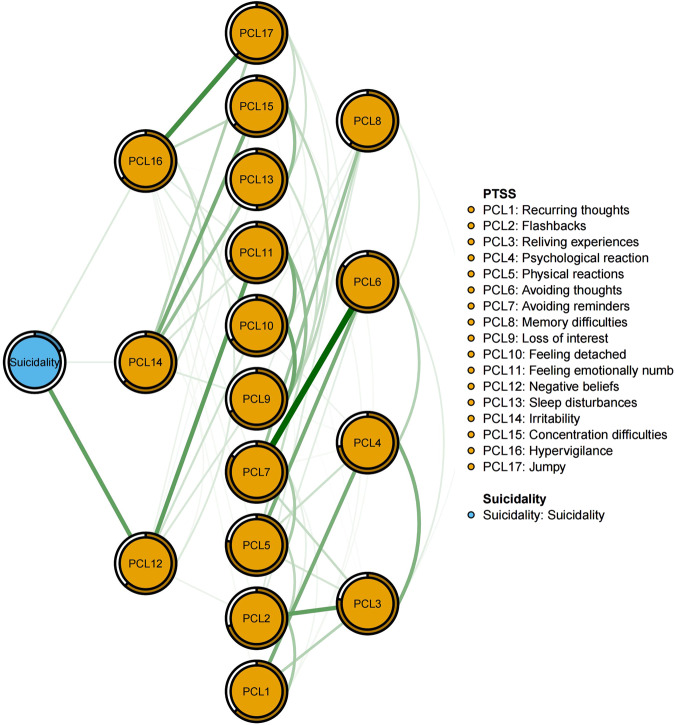
Flow network of suicidality and PTSS. Green edges represent positive partial correlations.

Figure 3 indicates PCL12 (“Negative belief”; average edge weight = 0.319), PCL 16 (“Hypervigilance”; average edge weight = 0.070) and PCL 14 (“Irritability”; average edge weight = 0.054) were nodes having the strongest, direct positive associations with suicidality. These three symptoms were members of the “Avoidance/Numbing” (PCL12) and Hyperarousal (PCL16, PCL14) dimensions.

## Discussion

To the best of our knowledge, this is the first study to examine the epidemiology of PTSS among MHPs immediately after China’s the “Dynamic Zero-COVID Policy” was terminated. The prevalence of PTSS among MHPs was 6.7% (95% CI = 6.2–7.2%). This rate is higher than the prevalence among Wuhan residents (5.9%; 95% CI = 5.0–6.8%) [48] and home-quarantined Chinese university students (2.7%) [49] but lower than the rate for COVID-19 survivor patients (18.7%; 95%CI = 12.0–25.3%) [50], all of which were assessed using the same PCL-C cut-off value, after the initial COVID-19 pandemic stage. This finding underscores how MHPs are at risk for lingering PTSS compared to general samples, except for survivors more directly affected by COVID-19.

However, the current PTSS prevalence for MHWs appears to be lower than rates in previous investigations of HCWs. This discrepancy may be a partial reflection of between study differences in PTSS measurement tools, sample experiences with COVID-19 exposure, pandemic phase, and regions assessed [50]. For example, one study reported a PTSS prevalence of 31.6% among HCWs dispatched to Wuhan during the initial peak of the pandemic based on Impact of Event Scale-Revised (IES-R) scores [51] while other studies found rates of 13.7% among Wuhan HCWs as measured by PCL-5 [14] and 49.3% among Italian HCWs based on Global Psycho-trauma Screen (GPS) scores [52]. Notably, PTSS among MHPs, as providers of mental health services, are susceptible to being overlooked compared to PTSS among HCWs involved in direct medical care to address COVID-19.

Sociodemographic correlates of PTSS included a married relationship status, as well as lower economic status and health status, in line with prior studies [45, 53]. Although being married often has protective health effects, in this study the higher PTSS level of married versus unmarried MHW subgroups may have been due to increased concerns about the health of one’s family during the pandemic such as fear of family members being infected, especially vulnerable children [53] or parents.

The PTSS subgroup difference for health status is consistent with evidence that individuals with perceived poor health status report more stress [54]. Ongoing stress can induce immune function dysregulation (i.e. increased blood levels of pro-inflammatory cytokines) and result in worsened symptoms of physical and psychiatric illnesses [55]. Furthermore, the PTSS subgroup difference in reported economic status aligns with research indicating economic strain in the context of COVID-19 is related to increased PTSS [56, 57]. Income levels of MHPs may have been reduced due to overall decrease in hospital revenues during the COVID-19 era [58]. Participants in our study, whose mean age was 34.85 years, were likely to be primary contributors to household income so financial stress induced by lockdowns may have had negative repercussions for mental health [59]. Thus, in the context of continual traumatic exposure, more physically and economically vulnerable MHPs likely experienced greater vulnerability to PTSS.

Per our results, quarantine experience during the COVID-19 pandemic is another risk factor for psychological problems [45]. Quarantined people experience negative emotions such as fear, nervousness, sadness and guilt due to isolation and loss of autonomy [60]. As previously highlighted, socialization and connectivity play an important role in maintaining physical and psychological well-being [61]. Under the “Dynamic Zero-COVID” policy, quarantining potential infection sources was an effective way of physically blocking the spread of the pandemic [2]. However, a sense of isolation, neglect, and loneliness are concurrent psychological effects of quarantines [62]. Quarantined persons can also report stigma and rejection from people in their local neighborhoods [46]. Discrimination and rejection experienced by quarantined MHPs could have contributed to their heightened risk for PTSS [63]. Uncertainty about whether they will recover fully after infection is another factor that can increase risk for PTSS among quarantined MHPs [50]. Finally, avoidance is a key characteristic of PTSS. Previous research revealed that being quarantined was associated with avoidance behaviors among HCWs such as avoiding direct contact with patients [64]. During the SARS period, 54% of quarantined people avoided others who were coughing or sneezing and 21% avoided all public places in the weeks that followed quarantines [60].

PTSD is often accompanied by other psychiatric problems [65, 66]. Strong relationships between depression and PTSS have been found in previous studies and are viewed as bidirectional in nature [10, 50, 51]. Depression was one of the most common psychological reactions among HCWs during earlier COVID-19 pandemic phases [67] and can increase PTSS risk for some [68]. Conversely, distress caused by PTSS such as intrusion and avoidance symptoms could trigger or exacerbate depression [50, 69]. As well, overlaps between PCL-C and PHQ-9 items (i.e., loss of interest, sleep disturbances, concentration problems, suicidal ideation) could magnify associations between depression and PTSS [50]. Depression and PTSS also have shared neurobiological underpinnings including reduced synaptic density and network-level alterations associated with increased severity of symptoms [70]. In light of these findings, antidepressant treatments may be a useful complementary therapy for PTSS [71].

In the PTSS network, “Avoiding thoughts” (PCL6), “Avoiding reminders” (PCL7), and “Feeling emotionally numb” (PCL11) were the most central symptoms; these central symptoms are all members of the PTSS “Avoidance/Numbing” dimension and aligned with centrality results from a previous network analysis on MHPs during the “Dynamic Zero-COVID Policy” [24] and a general population study [48]. Nonetheless, PTSS network studies of male firefighters [34] and children/adolescents [72] have reported central symptoms from other PTSS dimensions. Therefore, interventions should be targeted depending on specific characteristics of particular populations under examination.

Avoidance may be a common coping approach for HCWs [73]. In our study, avoiding thoughts and reminders referred to cognitions, feelings, activities or situations that would remind the person of traumatic experience (i.e., COVID-19 and COVID-19-related prevention and control measures). To some extent, avoidance coping may provide short-term psychological protection and alleviate distress [74]. However, avoidance coping is also a significant predictor of PTSD onset [75] and chronic PTSD [75, 76]. In addition, long-term emotional suppression in response to stressful situations correlates with poor physical and psychological health outcomes [74].

“Feeling emotionally numb” was also a central symptom in our study and reflects a deficiency in the ability to respond to stressful events emotionally [9, 77]. Numbing in response to traumatic stressors can predict later PTSD and poor recovery of PTSD [78]. MHPs are susceptible to burnout and compassion fatigue due to heavy workloads, especially during pandemics [79, 80]. Ongoing worry about patients is a common reaction when working in highly stressful hospital environments and cumulative compassion fatigue may result in emotional exhaustion [81]. In addition, emotional numbing is an overlapping symptom of major depressive disorder and PTSD that may contribute to high comorbidity of these syndromes [78].

A growing body of studies has demonstrated a positive relationship between suicidality and PTSD [82–84]. Prior or current suicidal ideation (i.e., thoughts of ending one’s life) and suicide attempts are associated with a diagnosis of PTSD [83, 84]. In addition, PTSD is related to an increased frequency of suicidality [82, 85]. In the PTSS and suicidality network, “Negative belief” (PCL12) had the strongest association with suicidality and refers to feeling that the future would somehow be cut short or the absence of future expectations [31]. Hyper-arousal symptoms (PCL16: “Hyper-vigilance”; PCL14: “Irritability”) were also strongly associated with suicidality in line with prior studies [86, 87]. Briere, et al [87]. found hyper-arousal symptoms fully mediated the relationship between exposure to traumatic events and severity of suicidality compared to other PTSS dimensions (e.g., intrusion and avoidance). Thus, alleviating these hyper-arousal symptoms among MHPs via interventions such as cognitive processing therapy [88] warrants consideration as a means of reducing suicidality among at-risk persons.

The merits of this study included a recent large sample size based on a national survey, and use of traditional analysis methods in tandem with network analysis to highlight predictors of overall PTSS and particularly critical symptoms, respectively. Several limitations should also be noted. First, causal relationships between variables could not be determined due to the cross-sectional study design. Second, assessments were based on self-report scales (e.g. PHQ-9, PCL-C) that may increase risk for recall and social desirability biases. Third, findings reflect PTSS rather than a PTSD diagnosis because structured diagnostic interviews (e.g., DSM-5) were not used. Fourth, following previous investigations run during the COVID-19 pandemic [25–27], this study was conducted online based on a convenience sampling method. Thus, selection biases may have affected the data. Finally, the gender imbalance (female: 82%) in our study was in line with the overall gender distribution of HCWs in China, but was slightly higher than that of Chinese HCWs (female: 74.4%) [89]. Consequently, our results may apply more strongly to female than male MHPs.

In conclusion, a high prevalence of lingering PTSS was found among MHPs immediately after China’s “Dynamic Zero-COVID Policy” terminated. At risk cohorts were more likely to be married, have had quarantine experience, report a poor financial status and health status, experience suicidality and more severe depressive symptoms. Avoidance/Numbing symptoms were most central in the PTSS network and included “Avoiding thoughts”, “Avoiding reminders” and “Feeling emotionally numbing”. Aside from these symptoms, “Negative Beliefs about the future” and hyper-arousal symptoms (e.g., “Hyper-vigilance and “Irritability”) warrant attention as specific targets for interventions to treat PTSS and prevent suicide among at-risk MHPs.

### Supplementary information

Supplementary materials

## Data Availability

The datasets presented in this article are not readily available because the Institutional Review Board of the Beijing Anding Hospital that approved the study prohibits the authors from making publicly available the research dataset of clinical studies. Requests to access the datasets should be directed to xyutly@gmail.com.
