# Supplementary material for: Prevalence and network structure of post-traumatic stress symptoms and their association with suicidality among Chinese mental health professionals immediately following the end of China’s Dynamic Zero-COVID Policy: a national survey
Source: Transl Psychiatry. 2023 Dec 15;13:395. doi: 10.1038/s41398-023-02680-3 (PMC10724192; doi:10.1038/s41398-023-02680-3)
Supplement: Supplementary file 1 — Supplementary materials [file 41398_2023_2680_MOESM1_ESM.docx]

**Supplementary materials**

Figure S1. Network stability of PTSS among Chinese mental health professionals

Figure S2. Estimation of edge weight difference by bootstrapped difference test

Figure S3. Bootstrapped confidence intervals of edge weights

Figure S4. Comparisons of network properties between the samples with at least 1-week quarantine experience (n=5,873) and those without quarantine experience (n=4,774)

Table S1. Descriptive information and network centrality indices of PTSS

| Item | Item content | Mean (SD) | Predictability | EI |
| --- | --- | --- | --- | --- |
| PCL1 | Recurring thoughts | 1.34 (0.658) | 0.631 | 0.789 |
| PCL2 | Flashbacks | 1.20 (0.535) | 0.707 | 0.879 |
| PCL3 | Reliving experiences | 1.23 (0.572) | 0.774 | 1.054 |
| PCL4 | Psychological reaction | 1.30 (0.647) | 0.729 | 0.941 |
| PCL5 | Physical reactions | 1.19 (0.543) | 0.772 | 0.922 |
| PCL6 | Avoiding thoughts | 1.21 (0.560) | 0.843 | 1.189 |
| PCL7 | Avoiding reminders | 1.20 (0.557) | 0.843 | 1.157 |
| PCL8 | Memory difficulties | 1.23 (0.553) | 0.602 | 0.670 |
| PCL9 | Loss of interest | 1.45 (0.751) | 0.677 | 1.032 |
| PCL10 | Feeling detached | 1.35 (0.689) | 0.667 | 0.949 |
| PCL11 | Feeling emotionally numb | 1.36 (0.720) | 0.702 | 1.074 |
| PCL12 | Negative beliefs | 1.29 (0.680) | 0.607 | 0.822 |
| PCL13 | Sleep disturbances | 1.74 (0.909) | 0.498 | 0.718 |
| PCL14 | Irritability | 1.64 (0.855) | 0.63 | 0.969 |
| PCL15 | Concentration difficulties | 1.58 (0.802) | 0.638 | 0.994 |
| PCL16 | Hypervigilance | 1.38 (0.713) | 0.648 | 0.963 |
| PCL17 | Jumpy | 1.50 (0.782) | 0.618 | 0.912 |

Note: SD: standard deviation; EI: Expected influence.


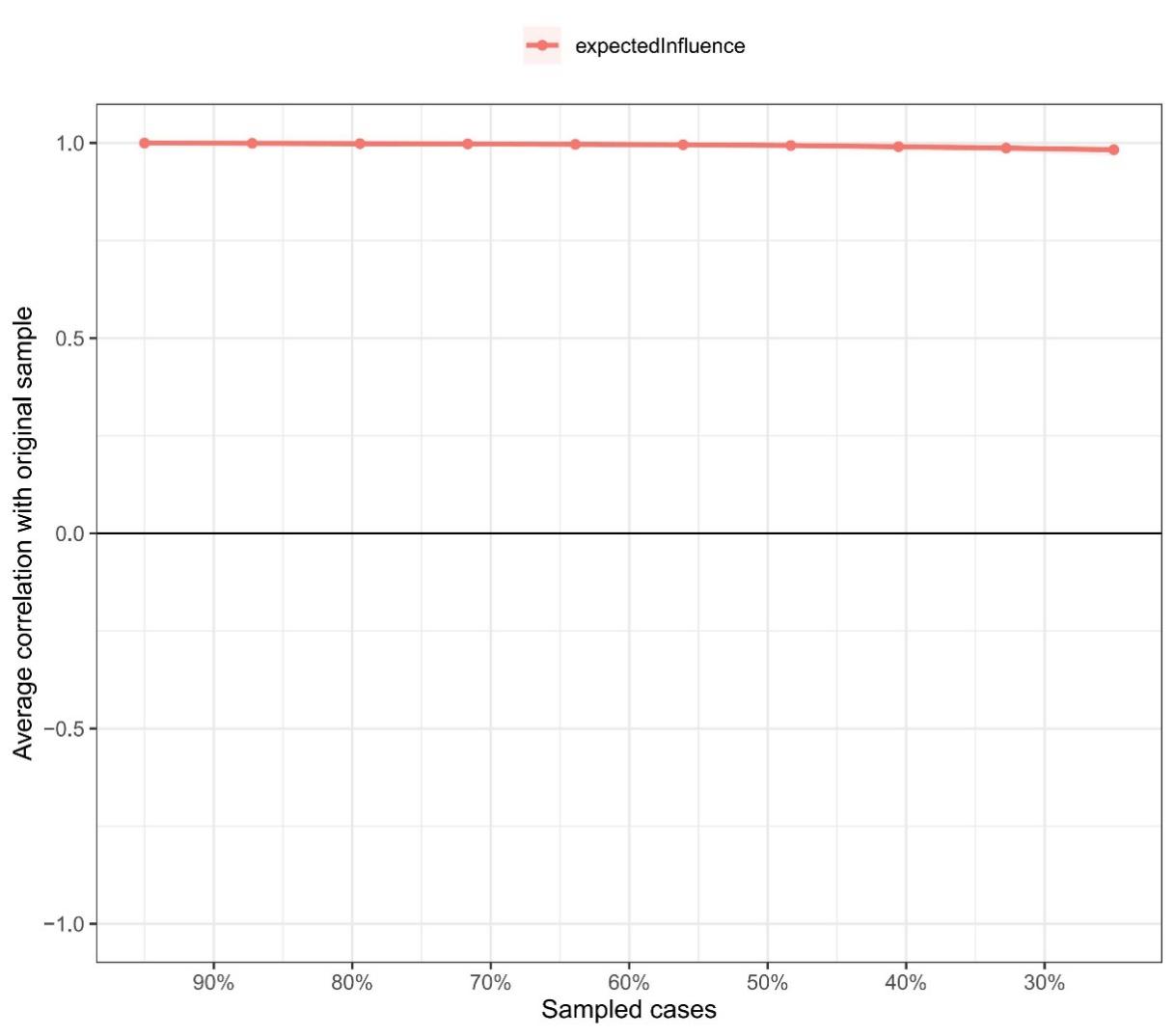


Figure S1. Network stability of PTSS among Chinese mental health professionals


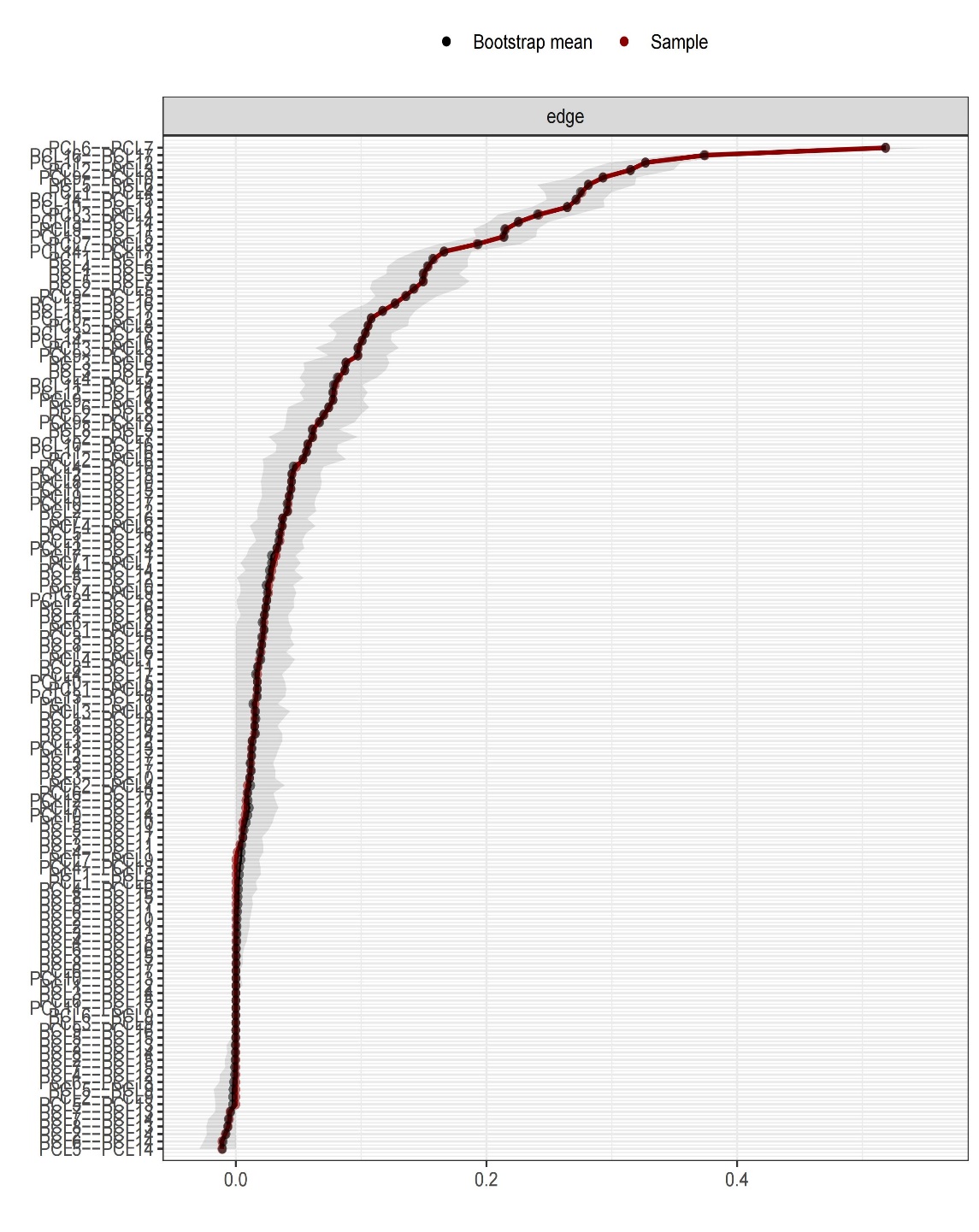


Figure S2. Estimation of edge weight difference by bootstrapped difference test

(Notes: The black dots indicate the values of each edge weight, ordered from the highest to the lowest value. The gray area represents the 95% confidence intervals of edge weights, estimated with the non-parametric bootstrap procedure)


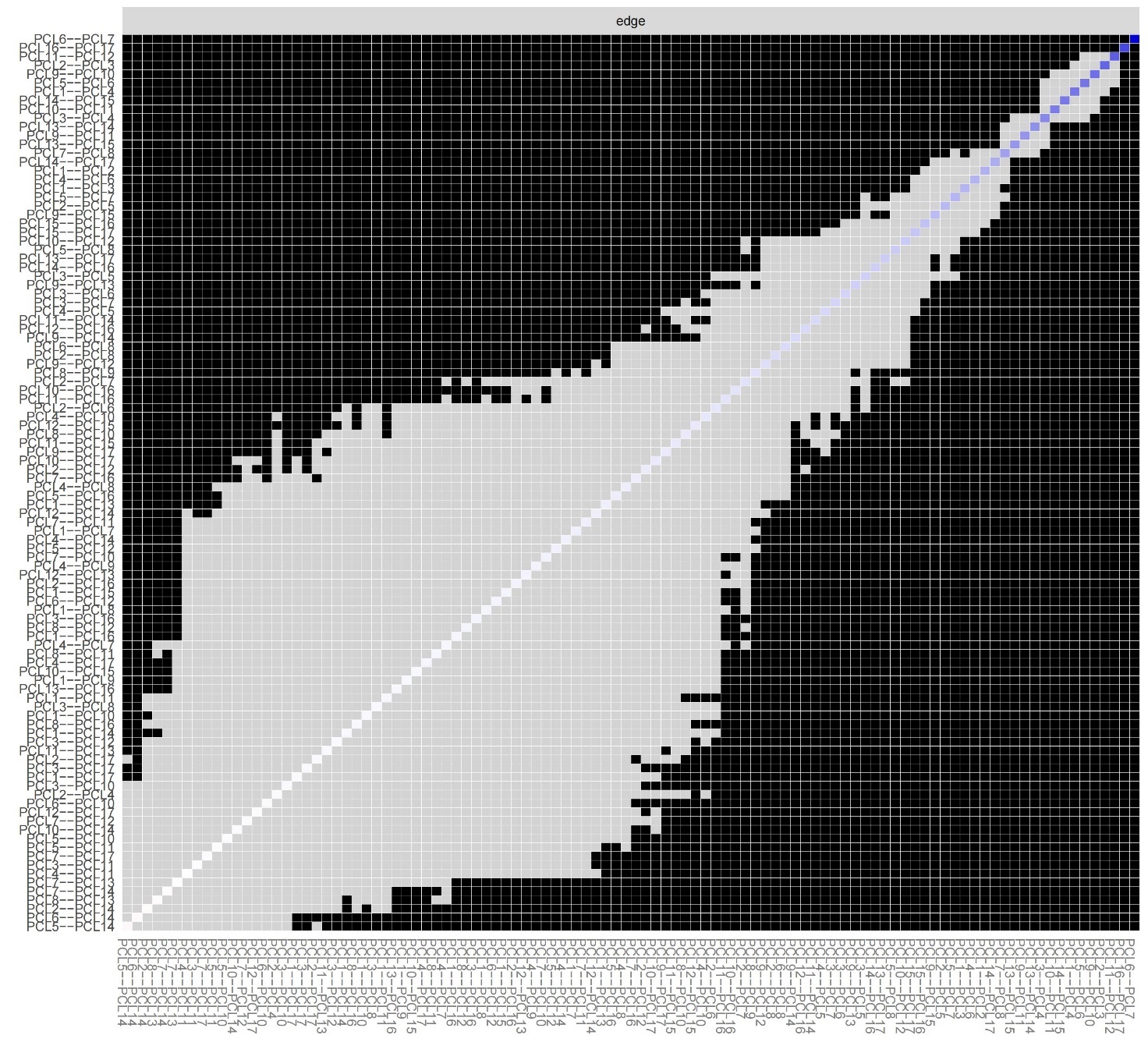


Figure S3. Bootstrapped confidence intervals of edge weights

(Notes: Gray boxes indicate edges that do not significantly differ from one-another. Black boxes represent edges with significant difference from one another (α = 0.05). Blue boxes in the edge-weight plot indicate positive correlations)


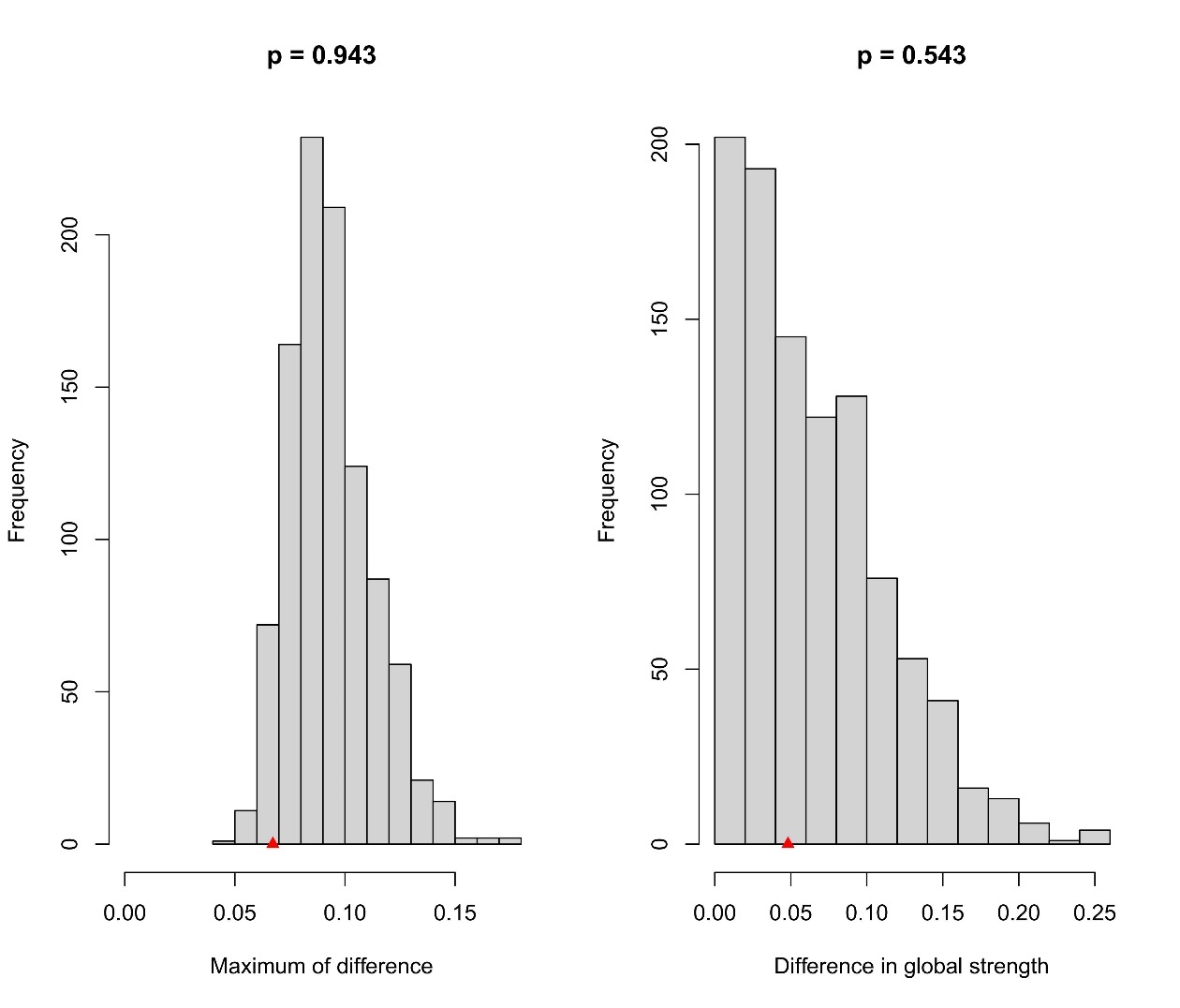


Figure S4. Comparisons of network properties between the samples with at least 1-week quarantine experience (n=5,873) and those without quarantine experience (n=4,774)
